# ATOMIX benchmark datasets for dissipation rate measurements using shear probes

**DOI:** 10.1038/s41597-024-03323-y

**Published:** 2024-05-21

**Authors:** Ilker Fer, Marcus Dengler, Peter Holtermann, Arnaud Le Boyer, Rolf Lueck

**Affiliations:** 1 Geophysical Institute, University of Bergen, Bergen, Norway; 2 GEOMAR Helmholtz Centre for Ocean Research Kiel, Kiel, Germany; 3 Leibniz Institute for Baltic Sea Research, Warnemünde, Rostock Germany; 4 Scripps Institution of Oceanography, University of California San Diego, San Diego, CA USA; 5 Rockland Scientific, Inc., Victoria, British Columbia Canada

**Keywords:** Physical oceanography, Fluid dynamics

## Abstract

Turbulent mixing in the ocean, lakes and reservoirs facilitates the transport of momentum, heat, nutrients, and other passive tracers. Turbulent fluxes are proportional to the rate of turbulent kinetic energy dissipation per unit mass, *ε*. A common method for *ε* measurements is using microstructure profilers with shear probes. Such measurements are now widespread, and a non-expert practitioner will benefit from best practice guidelines and benchmark datasets. As a part of the Scientific Committee on Oceanographic Research (SCOR) working group on “Analysing ocean turbulence observations to quantify mixing” (ATOMIX), we compiled a collection of five benchmark data of *ε* from measurements of turbulence shear using shear probes. The datasets are processed using the ATOMIX recommendations for best practices documented separately. Here, we describe and validate the datasets. The benchmark collection is from different types of instruments and covers a wide range of environmental conditions. These datasets serve to guide the users to test their *ε* estimation methods and quality-assurance metrics, and to standardize their data for archiving.

## Background & Summary

Turbulent mixing in the ocean plays a crucial role in regulating Earth’s climate by influencing the transport and distribution of heat, nutrients, and other solutes within the ocean. Ocean mixing can be quantified through turbulent fluxes across density surfaces, called the diapycnal fluxes, which were recently proposed as a pilot Essential Ocean Variable in the Global Ocean Observing System^1^. The diapycnal fluxes can be calculated using the rate of turbulent kinetic energy dissipation per unit mass, *ε* along with the background property gradients. The dissipation rates are typically measured by shear probes at small scales (${\mathcal{O}}(1{\rm{m}})-{\mathcal{O}}(1{\rm{cm}})$).

Despite the need for specialized instruments and complex procedures of data processing, microstructure shear probe measurements are now widespread, and a practitioner’s expertise may cover a broad range from a beginner to an expert user. With the motivation to provide data processing and quality-assurance guidelines as well as a standardized data format in the form of “best practices”, a Working Group on “Analyzing ocean turbulence observations to quantify mixing” (ATOMIX) was established under the Scientific Committee on Oceanographic Research (SCOR). SCOR is an international, non-governmental, non-profit organization. Since its establishment in 1957, SCOR has united ocean scientists from around the globe to advance the field of ocean science. Scientific working groups within SCOR are formed by international members and are approved through a competitive assessment process. These groups focus on specific, narrow scientific topics to drive progress in the field. One objective of ATOMIX is to establish an open-access database of benchmark datasets that can be used to assess and validate algorithms for estimating *ε*, irrespective of the specific implementation in a programming language. Three subgroups in ATOMIX deal with three different approaches for dissipation estimates, each requiring a different measurement technology using shear probes, acoustic Doppler current profilers, and point-velocity measurements. This paper describes the *ε* benchmark datasets from shear probes. More details can be found on the wiki site of ATOMIX (https://wiki.app.uib.no/atomix).

**Fig. 1:**
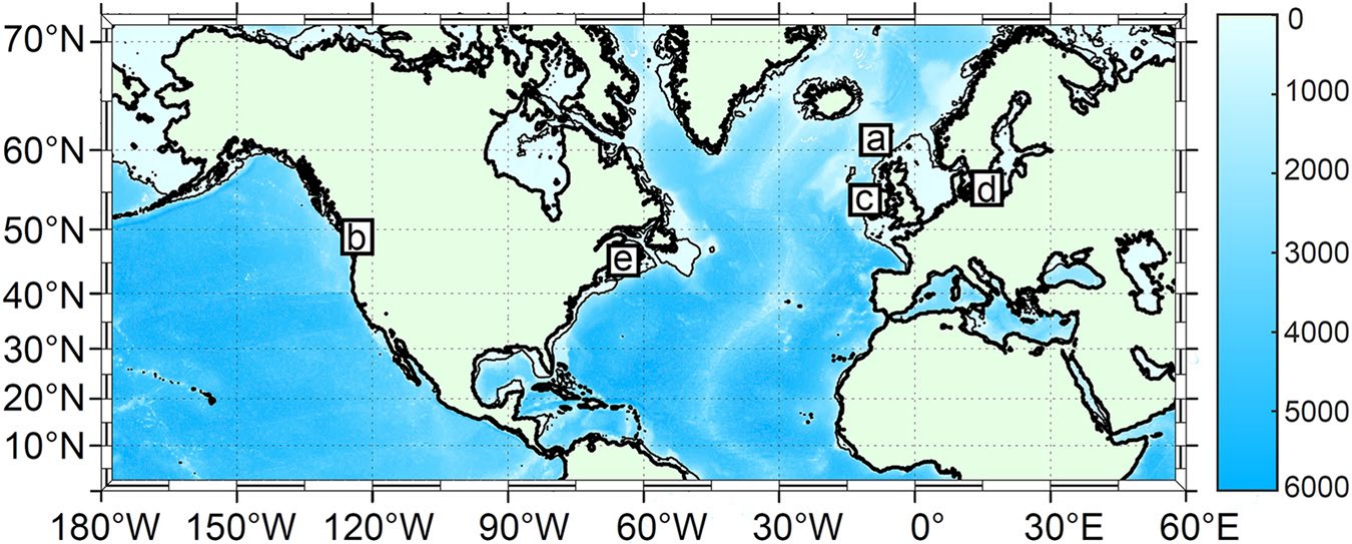
Map showing the locations where benchmark data sets were collected (squares). Letters indicate location of (**a**) Faroe Bank Channel^3^, (**b**) tidal channel adjacent to Haro Strait^4^, (**c**) Rockall Trough^5^, (**d**) Bornholm Basin in the Baltic Sea^6^ and (**e**) Minas Passage^7^. Shading is bathymetry^15^, downloaded from https://topex.ucsd.edu/marine_topo/mar_topo.html. The 200-m isobath is shown as a thin solid black line.

The shear probes group has provided a detailed description of ATOMIX best practices for data processing in a methods paper^2^. While the methods paper serves as a valuable resource for informing novice users about data processing, the objectives of the current paper are to describe and technically validate the benchmark datasets, offer a means for testing processing routines and define a standardized format for archiving the data. Lueck *et al*.^2^ used a dissipation profile from the Faroe Bank Channel^3^–one of the five benchmark datasets reported here–as an example to discuss various choices in data processing and quality assurance. In this paper, we describe and technically validate a collection of five benchmark datasets^3–7^. We also provide detailed information on the data file format, variables, and content. The datasets come from various sites (Fig. 1, Table 1) and are selected to cover a broad range of depths, flow dynamics, and sampling strategies using different platforms and instruments for data collection.

Four sets are from vertical profilers, two manufactured by Rockland Scientific, Canada, one by Sea & Sun Technology, Germany, and one developed at Scripps Institution of Oceanography, USA. The fifth dataset is from a mooring equipped with a turbulence recording package manufactured by Rockland Scientific, Canada. These instruments cover most of those that are available to the community and the benchmarks showcase various aspects of dissipation rate estimates. Each dataset is presented in a documented, homogeneous format, encompassing all defined levels of the ATOMIX format. Each dataset is compared and validated by using different data processing routines of the ATOMIX members involved in this study. These datasets (*i*) show how shear-probe data are processed to derive the rate of dissipation, *ε*, (*ii*) provide a means for researchers to assess and evaluate their own processing routines and, (*iii*) (because of the wide range of instruments in the dataset) furnish a resource for developing the platform-independent analysis of shear-probe data.

## Methods

The benchmark datasets described here are processed following the best practices recommendations from ATOMIX, which are documented in detail in Lueck *et al*.^2^ and on the wiki site https://wiki.app.uib.no/atomix/. A user must refer to the methods paper^2^ for details on processing shear-probe data, and be familiar with considerations for parameter choices and quality-assurance metrics. In summary, the recommended procedure has four levels, and the data structure format reflects these levels. In Level 1, the data are converted into physical units of shear. When an instrument carries two shear probes, it is good practice to orient them to sense two orthogonal components of shear so that there are two statistically independent estimates of the rate of dissipation, *ε* (e.g., the Faroe Bank Channel profile^3^). When the sensing axis of a probe is not known, the shear probes are mounted in random orientation (e.g., the Baltic Sea profile^6^); however the dissipation estimates are similar under the local isotropy assumption. When there are more than two probes (e.g., the Minas Passage profile^7^), some of them can be oriented to sense the same component of shear which provides measurement redundancy and a backup in case of probe failure. In Level 2, the time series are prepared in sections for analysis. A “section” is defined as a continuous part of a time series that satisfies certain criteria and is long enough to provide at least one estimate of the rate of dissipation. The record from each section is de-spiked and high-pass filtered to delineate the scales of interest which are determined by the length of the instrument and the rate of dissipation itself. Level 3 is the spectral calculations resulting in wavenumber spectra of shear from which, typically, the vibration-coherent contamination is removed. Finally, in Level 4, the rates of dissipation, *ε*, are estimated from the spectra of shear together with the quality-assurance metrics. When dissipation estimates are available from more than one probe, the final estimate is the average of those estimates that satisfy the quality assurance criteria. These levels are abbreviated L1, L2, L3, and L4, respectively. For ${\varepsilon }\lesssim 1{0}^{-5}{{\rm{W}}{\rm{k}}{\rm{g}}}^{-1}$, we use the method of spectral integration after carefully choosing the upper and lower wavenumber limits of integration. A model spectrum such as Nasmyth, Lueck or Panchev-Kesich is then used to correct for the fraction of the variance that is excluded by the integration limits. For high dissipation rates (${\varepsilon }\gtrsim 1{0}^{-5}{{\rm{W}}{\rm{k}}{\rm{g}}}^{-1}$), we estimate *ε* by fitting to the inertial subrange of the model spectrum. More details on the choice of processing parameters for each dataset are given in the specific descriptions below ε.

The processing of shear-probe data involves many parameters and the appropriate values of these parameters are discussed in Lueck *et al*.^2^. The values of the parameters that were chosen for the benchmark datasets are appropriate for the scientific purposes for which these data were collected, but they may not be the only good choice. The user can also examine how differing values for the processing parameters affect the dissipation estimates. Processing always involves a compromise between the statistical reliability of an *ε* estimate (which improves with increasing data length used for the estimate) and the spatial resolution of the estimates (which becomes finer with decreasing data length). When analyzing their data, users are strongly encouraged to implement the ATOMIX recommendations in choosing the processing parameters for their specific instrument, sampling strategy and environment.

### Description of the ATOMIX format

The datasets are prepared as Network Common Data Form (netCDF) files in a homogeneous format that includes four hierarchical groups, corresponding to the four processing levels. The group names are L1_converted, L2_cleaned, L3_spectra and L4_dissipation, corresponding to L1 to L4. Attributes are provided both at the global level and at the group level. Dimension names and their description at each level are summarized in Table 2. Detailed lists of variables for each level, together with standard names, units and dimensions, are given in Table 3 for L1, Table 4 for L2, Table 5 for L3, and Table 6 for L4. The standard names follow the Climate and Forecast (CF) convention and their guidelines for the construction of standard names. A variable is identified as required (R), highly recommended (HR) or optional (O). This distinction is not based on a specific convention but is defined by the ATOMIX Working Group to ensure the variables needed to process shear probe data are provided. A list of required global attributes (Table 7) identifies the minimum metadata needed to describe the dataset and the choices of data processing. We recommend including the optional metadata (Table 8, and additional metadata defined by the user) to the extent possible. We strongly recommend duplicating both required and optional global attributes at their relevant group levels. For instance, attributes about sampling and profiling should be repeated at L1, those related to cleaning and de-spiking at L2, spectral calculations at L3, and dissipation estimates at L4.

**Table 1:** Overview of benchmark data.

Site	Instrument	Citation	DOI
Faroe Bank Channel	VMP2000	Fer^3^	10.5285/05f21d1d-bf9c-5549-e063-6c86abc0b846
Haro Strait	VMP250	Lueck^4^	10.5285/0ec16a65-abdf-2822-e063-6c86abc06533
Rockall Trough	Epsilometer	Le Boyer *et al*.^5^	10.5285/0ebffc86-ed32-5dde-e063-6c86abc08b3a
Baltic Sea	MSS90-L	Holtermann^6^	10.5285/0e35f96f-57e3-540b-e063-6c86abc06660
Minas Passage	MR1000	Lueck and Hay^7^	10.5285/0ec17274-7a64-2b28-e063-6c86abc0ee02

**Table 2:** NetCDF dimension names and description for shear probe data.

Dimension	Level	Description
TIME	L1	Length of the record from turbulence (fast) data channels
TIME_***^a^	L1	Length of the record from slow data channels (if different from fast)
N_SHEAR_SENSORS	L1	Number of shear channels (shear sensors)
N_***_SENSORS^*b*^	L1	Number of *** channels (sensors)
TIME	L2	Length of the record from turbulence (fast) data channels
N_SHEAR_SENSORS	L2	Number of shear channels (shear sensors)
N_***_SENSORS^*b*^	L2	Number of *** channels (sensors)
TIME_SPECTRA	L3	Length of the record of average times of spectral segments
N_WAVENUMBER	L3	Length of the wavenumber array
N_SHEAR_SENSORS	L3	Number of shear channels (shear sensors)
N_***_SENSORS^*b*^	L3	Number of *** channels
N_SH_ACC_SPEC	L3	Number of shear-acceleration cross spectra
N_SH_VIB_SPEC	L3	Number of shear-vibration cross spectra
N_GLOBAL_VALUES^*c*^	L3	Dimension for 1 data point (for the entire analysis)
TIME_SPECTRA	L4	Length of the record of average times of spectral segments
N_SHEAR_SENSORS	L4	Number of shear channels (shear sensors)

^*a*^Typically TIME is assumed for the fast-sampled microstructure channels. Use e.g., TIME_SLOW or TIME_CTD for slower sampled channels such as CTD and tilt sensors.

^*b*^Example dimension names would be: N_VIB_SENSORS for vibration (piezo-acceleration) sensors, N_ACC_SENSORS for vibration acceleration sensors.

^*c*^Dimension for variables of the size 1 × 1 for variables such as N_FFT_SEGMENTS and DOF.

**Table 3:** NetCDF variable names in the Level 1 group.

Variable name	Req.^*a*^	Standard name	Unit	Dimensions
TIME	R	time	CF-convention^*b*^	TIME
SHEAR	R	[sea_]^*c*^ water_velocity_shear	s-1	TIME, N_SHEAR_SENSORS
PSPD_REL	HR	platform_speed_wrt_[sea_]^*c*^ water	m s-1	TIME
VIB	HR	platform_vibration	—	TIME, N_VIB_SENSORS
PRES	HR	[sea_]^*c*^ water_pressure	dbar	TIME
TEMP	HR	[sea_]^*c*^ water_temperature	degree_Celsius	TIME, N_T_SENSORS
ACC	O	platform_acceleration	— or m s-2	TIME, N_ACC_SENSORS
CNDC	O	[sea_]^*c*^ water_electrical_conductivity	S m-1	TIME, N_C_SENSORS
GRADT	O	derivative_of_[sea_]^*c*^ water_temperature_wrt_*^d^	degree_Celsius m-1	TIME, N_GRADT_SENSORS
GRADC	O	derivative_of_[sea_]^*c*^ water_conductivity_wrt_*^d^	— or S m-2	TIME, N_GRADC_SENSORS
PITCH	O	platform_pitch_angle	degree	TIME
ROLL	O	platform_roll_angle	degree	TIME

^*a*^Code for the requirement of variable, R: Required, HR: Highly recommended, O: Optional.

^*b*^Unit and offset need to be compatible with the Climate and Forecast (CF)-convention.

^*c*^User can choose between water or sea_water depending on the environment.

^*d*^wrt_z or wrt_s; spatial derivative. Typically derived from the rate of change of temperature and divided by the profiling speed.

**Table 4:** NetCDF variable names in the Level 2 group.

Variable name	Req.^*a*^	Standard name	Unit	Dimensions
TIME	R	time	CF-Convention^*b*^	TIME
SHEAR	R	[sea_]^*c*^ water_velocity_shear	s-1	TIME, N_SHEAR_SENSORS
PSPD_REL	R	platform_speed_wrt_[sea_]^*c*^ water	m s-1	TIME
SECTION_NUMBER	R	unique_identifier_for_each_section_ of_data_from_timeseries	—	TIME
VIB	HR	platform_vibration	m s-2, or - ^*d*^	TIME, N_VIB_SENSORS
ACC	O	platform_acceleration	m s-2, or - ^*d*^	TIME, N_ACC_SENSORS

^*a*^Code for the requirement of variable, R: Required, HR: Highly recommended, O: Optional.

^*b*^Unit and offset need to be compatible with the Climate and Forecast (CF)-convention.

^*c*^User can choose between water or sea_water depending on the environment.

^*d*^Acceleration and vibration sensors are sometimes not calibrated and their records are used as raw values.

**Table 5:** NetCDF variable names in the Level 3 group.

Variable name	Req.^*a*^	Standard name	Unit	Dimensions
TIME	R	time	CF-Convention^*b*^	TIME_SPECTRA
SECTION_NUMBER	R	unique_identifier_for_each_section_ of_data_from_timeseries	—	TIME_SPECTRA
PSPD_REL	R	platform_speed_wrt_[sea_]^*c*^ water	m s-1	TIME_SPECTRA
SH_SPEC	R	shear_probe_spectrum	s-2 cpm-1	TIME_SPECTRA, N_WAVENUMBER, N_SHEAR_SENSORS
KCYC	R	cyclic_wavenumber	cpm	TIME_SPECTRA, N_WAVENUMBER
SH_SPEC_CLEAN	R	shear_probe_spectrum_clean	s-2 cpm-1	TIME_SPECTRA, N_WAVENUMBER, N_SHEAR_SENSORS
N_FFT_SEGMENTS	R	number_of_fft_segments	—	N_GLOBAL_VALUES
N_VIB_SENSORS	R	number_of_vibration_sensors_used_for_cleaning_spectra	—	N_GLOBAL_VALUES
SPEC_STD	R	standard_deviation_uncertainty_of_shear_spectrum	—	N_GLOBAL_VALUES
PRES	HR	[sea_]^*c*^ water_pressure	dbar	TIME_SPECTRA
ACC_SPEC	O	acceleration_sensor_spectrum	— or m2 s-4 cpm-1	TIME_SPECTRA, N_WAVENUMBER, N_ACCEL_SENSORS
VIB_SPEC	O	vibration_sensor_spectrum	—	TIME_SPECTRA, WAVENUMBER, N_VIB_SENSORS
SH_VIB_SPEC	O	shear_and_vibration_cross-spectral_matrix	—	TIME_SPECTRA, N_WAVENUMBER, N_SH_VIB_SPEC
SH_ACC_SPEC	O	shear_and_acceleration_cross-spectral_matrix	—	TIME_SPECTRA, N_WAVENUMBER, N_SH_ACC_SPEC
DOF	O	degrees_of_freedom_of_spectrum	—	N_GLOBAL_VALUES

^*a*^Code for the requirement of variable, R: Required, HR: Highly recommended, O: Optional.

^*b*^Unit and offset need to be compatible with the Climate and Forecast (CF)-convention.

^*c*^User can choose between water or sea_water depending on the environment.

**Table 6:** NetCDF variable names in the Level 4 group.

Variable name	Req.^*a*^	Standard name	Unit	Dimensions
TIME	R	time	CF-Convention^*b*^	TIME_SPECTRA
SECTION_NUMBER	R	unique_identifier_for_each_section_ of_data_from_timeseries	—	TIME_SPECTRA
PSPD_REL	R	platform_speed_wrt_[sea_]^*c*^ water	m s-1	TIME_SPECTRA
EPSI	R	specific_turbulent_kinetic_energy_dissipation_in_[sea_]^*c*^ water	W kg-1	TIME_SPECTRA, N_SHEAR_SENSORS
EPSI_FINAL	R	specific_turbulent_kinetic_energy_dissipation_in_[sea_]^*c*^ water	W kg-1	TIME_SPECTRA
KMIN	R	minimum_wavenumber_used_for_estimating_turbulent_kinetic_energy_dissipation	cpm	TIME_SPECTRA, N_SHEAR_SENSORS
KMAX	R	maximum_wavenumber_used_for_estimating_turbulent_kinetic_energy_dissipation	cpm	TIME_SPECTRA, N_SHEAR_SENSORS
N_S	R	number_of_spectral_points_used_for_estimating_turbulent_kinetic_energy_dissipation	—	TIME_SPECTRA, N_SHEAR_SENSORS
EPSI_STD	R	expected_standard_deviation_of_the_logarithm_of_the_dissipation_estimate	—	TIME_SPECTRA, N_SHEAR_SENSORS
EPSI_FLAGS	R	dissipation_qc_flags	—	TIME_SPECTRA, N_SHEAR_SENSORS
METHOD	R	method_used_for_estimating_turbulent_kinetic_energy_dissipation	—	TIME_SPECTRA, N_SHEAR_SENSORS
PRES	HR	[sea_]^*c*^ water_pressure	dbar	TIME_SPECTRA
KVISC	HR	kinematic_viscosity_of_[sea_]^*c*^ water	m2 s-1	TIME_SPECTRA
FOM	HR	figure_of_merit	—	TIME_SPECTRA, N_SHEAR_SENSORS
MAD	O	mean_absolute_deviation	—	TIME_SPECTRA, N_SHEAR_SENSORS
VAR_RESOLVED	O	variance_resolved	—	TIME_SPECTRA, N_SHEAR_SENSORS
DESPIKE_FRACTION_SH	O	fraction_of_shear_data_modified_by_despiking_algorithm	—	TIME_SPECTRA, N_SHEAR_SENSORS
DESPIKE_PASS_COUNT_SH	O	number_of_despike_passes_for_shear_probes	—	TIME_SPECTRA, N_SHEAR_SENSORS

^*a*^Code for the requirement of variable, R: Required, HR: Highly recommended, O: Optional.

^*b*^Unit and offset need to be compatible with the Climate and Forecast (CF)-convention.

^*c*^User can choose between water or sea_water depending on the environment.

**Table 7:** Global attributes: required metadata.

Attribute Name	Description	Convention
title	A comprehensive title for the dataset including the time and location aspect	CF, ACDD
authors	A list of authors	ATOMIX
summary	An abstract describing the dataset	ACDD
comment	Supplementary technical details about the collecting and processing of the dataset	CF, ACDD
platform	The platform from which the data are collected. e.g. sub-surface mooring, research vessel, sub-surface glider	ACDD
source	The instrument used for collecting the data. For example, vertical microstructure profiler, VMP2000 SN009.	CF, ACDD
date_created	The date on which the data were created, yyyy-mm-ddTHH:MM:SSZ	ACDD
date_modified	The date on which the data were last modified, yyyy-mm-ddTHH:MM:SSZ	ACDD
time_reference_year	Year for time reference	ATOMIX
time_coverage_start	Time of the first data point in the dataset, yyyy-mm-ddTHH:MM:SSZ	ACDD
time_coverage_end	Time of the last data point in the dataset, yyyy-mm-ddTHH:MM:SSZ	ACDD
geospatial_lat_min	Southern bound of data, decimal degrees, negative for South	ACDD
geospatial_lat_max	Northern bound of data, decimal degrees, negative for South	ACDD
geospatial_lon_min	Western bound of data, decimal degrees, negative for West	ACDD
geospatial_lon_max	Eastern bound of data, decimal degrees, negative for West	ACDD
fs_fast	Sampling frequency for fast (turbulence) channels	ATOMIX
fs_slow	Sampling frequency for slow channels (if exists). Alternative names could be, e.g., fs_ctd	ATOMIX
profiling_direction	Direction along which the section was collected, e.g., horizontal, vertical, or glide	ATOMIX
fft_length	Length of the Fast Fourier transform segments (in data points; note, fft_lengths_sec in seconds is optional)	ATOMIX
diss_length	Length of data (in data points) used for each dissipation estimate	ATOMIX
overlap	Length of overlap (in data points) in diss_length	ATOMIX
goodman	Flag for the vibration coherent noise removal using the Goodman algorithm. 0 = not applied; 1 = applied	ATOMIX
HP_cut	The high-pass filter cutoff frequency in Hz. Can be zero for no filtering	ATOMIX
conventions	A comma-separated list of the conventions that are followed by the dataset. e.g., CF-1.6, ACDD-1.3, ATOMIX-1.0	CF, ACDD
history	Provides an audit trail for modifications to the original data; e.g., Version 1	CF, ACDD

It is highly recommended to duplicate relevant attributes at the corresponding group level. Attributes not listed in the Climate and Forecast (CF) and the Attribute Convention for Data Discovery (ACDD) standards are labeled as ATOMIX.

**Table 8:** Global attributes: optional metadata.

Attribute Name	Description	Convention
fft_length_sec	Length of the Fast Fourier transform segments in seconds	ATOMIX
diss_length_sec	Dissipation estimate data length in seconds	ATOMIX
overlap_sec	Length of overlap (in seconds) in diss_length_sec	ATOMIX
f_AA	The anti-aliasing frequency in Hz	ATOMIX
FOM_limit	Figure of merit limit for quality assurance. Typically between 1.15 and 1.4	ATOMIX
diss_ratio_limit	The limit to identify anomalously large disagreement between dissipation estimates from probes. The magnitude of the difference of the natural logarithm of two dissipation estimates should be smaller than diss_ratio_limit ×*σ*_In*ε*_(EPSI_STD). Typically, 2.77	ATOMIX
despike_shear_fraction_limit	The maximum allowed fraction of data (of each diss_length_sec length) removed by de-spiking. Typically, 0.05	ATOMIX
despike_shear_iterations_limit	The maximum number of allowed iterations of de-spiking when producing the L2 shear probe data (one value per section). Typically 8	ATOMIX
variance_resolved_limit	The minimum fraction of variance resolved in an estimate by spectral integration. Typically 0.6	ATOMIX
f_limit	The upper limit to exclude frequencies from analysis. Typically infinity	ATOMIX
fit_2_isr	Dissipation threshold for using the method of fitting in the inertial subrange. Typically 10^−5^ W kg^−1^	ATOMIX
spectral_model	The model shear spectrum used in dissipation estimates with the integration method: e.g., Nasmyth, Lueck or Panchev-Kesich	ATOMIX
area	The region where the data were collected, e.g., Arctic Ocean, Barents Sea	ATOMIX
geospatial_vertical_min	Further refinement of the geospatial bounding box. Vertical minimum in m	ACDD
geospatial_vertical_max	Further refinement of the geospatial bounding box. Vertical maximum in m	ACDD
geospatial_vertical_positive	Direction of positive vertical: down, up	ACDD
institution	The name of the institution principally responsible for originating this data	CF, ACDD
principal_investigator	Name of the principal investigator who created the data	ATOMIX
contact	Name of the contact person	ACDD
project	The scientific project that produced the data	ACDD
cruise	The name or number of the research cruise	ATOMIX
vessel	The name of the research vessel	ATOMIX
references	A list of related references	CF, ACDD
keywords	A comma-separated list of keywords and phrases	ACDD
creator_name	The data creator’s name	ACDD
creator_email	The data creator’s email	ACDD
creator_url	The data creator’s URL	ACDD
acknowledgement	Acknowledgement of support for the project that produced this data	ACDD
station_name	The name of the station where data were collected	ATOMIX
license	Provide the URL to a standard or specific license, e.g, http://creativecommons.org/licenses/by/4.0/, Freely Distributed, or None	ACDD

It is highly recommended to duplicate relevant attributes at the corresponding group level. Attributes not listed in the Climate and Forecast (CF) and the Attribute Convention for Data Discovery (ACDD) standards are labeled as ATOMIX.

L1 records must have, at a minimum, the time series of shear probe data converted to a shear measurement in physical units together with the time of the data samples. We recommend that the original, complete records needed for the profiling speed estimate (e.g., pressure, ocean currents etc.) are provided in L1 too, as well as supporting time series such as temperature (needed for accurate molecular viscosity calculations), pitch and roll (if available, useful for examining the platform behaviour), and high-resolution platform vibration or acceleration records. While not strictly required in a dataset, the vibration or acceleration records are highly recommended to accompany shear probe measurements, as these can be used to effectively remove platform motion contamination from the shear probe spectra^2^. In Table 3, we also list optional variables that may be sampled in a typical microstructure profiler, such as conductivity for salinity measurements, and scalar gradients. While the record length dimension is identified by TIME, representing the fast-sampled channels such as the shear records, an instrument can sample different channels at slower rates, such as precision conductivity-temperature- depth (CTD) sensors or attitude sensors to monitor the kinematics of the instrument, which can be identified using a dimension name reflecting the channel or the sensor, such as TIME_SLOW, TIME_CTD or similar. Some instruments are equipped with auxiliary sensors such as dissolved oxygen, chlorophyll-a fluorescence or similar. While not all records are required for dissipation measurements–the topic of this Work Group–it is highly recommended to include and archive data from all sensors converted into physical units in the L1 group.

L2 records must identify the parts of the time series that are used for dissipation estimation by way of a “section number”. In L2, the shear records are suitably high-pass filtered and, if necessary, cleaned by de-spiking. Each data point in the L2 shear probe record is identified with a section number (equal to 1, 2 and so on; a data record can have a single section or multiple sections such as in the Rockall Trough dataset^5^). In addition, the L2 record must include the estimates or direct measurements of the relative speed of profiling, i.e., platform speed with respect to water, interpolated or measured at the measurement time of the shear probe data. The profiling speed for a vertical profiler (*W*) is typically estimated from the rate of change of pressure after smoothing by a low-pass filter. In environments where the background vertical velocity is a substantial fraction of the target fall rate of the profiler (e.g., the Tidal Channel dataset^4^), the rate of change of pressure must be used with caution. In the records from the moored instrument in the Minas Passage^7^, the speed of profiling (*U*) was measured by an acoustic current meter.

L3 records are wavenumber spectra calculated using the shear probe data identified in L2. The spectra are calculated using records of length “diss_length” in data points, or “diss_length_sec” in seconds, which covers a minimum of 3 half-overlapping Fast Fourier Transform (FFT) segments. At a minimum, the required variables are the time (average time over diss_length), the section number identifier for the spectrum, the profiling speed (average speed over diss_length, and used in converting frequency to wavenumber), the wavenumber, and the corresponding spectral values for each shear probe. Additionally, the standard deviation uncertainty of the shear spectrum must be provided (this is one value for the entire analysis, and depends on the choices of spectral analysis)^2^. The shear spectra must be corrected for the high-pass filter (if one was applied), the wavenumber response of the shear probe, and for any other instrument-dependent frequency response characteristics of the measurement electronics. If the instrument is equipped with acceleration or vibration sensors and these are used to remove contamination from the shear spectra, cleaned shear spectra are also required.

L4 records are the dissipation rate estimates and related parameters from the spectra in L3. The section number, time, and profiling speed (as well as optional variables such as pressure and temperature averaged over diss_length) are required together with dissipation estimates from all probes (EPSI). Furthermore, each EPSI estimate must be accompanied by the minimum and maximum wavenumber used (KMIN and KMAX), the number of spectral points used (N_S), the expected standard deviation of the logarithm of the dissipation estimate (EPSI_STD, σ_lnε_), the method used for the estimate (a METHOD value of 0 for integration or 1 for fitting in the inertial subrange), and quality control flag values. We recommend including optional variables relating to the quality control of a dissipation estimate (see Table 6). The quality-assurance metrics are combined into a single flag value (*Q*, variable name EPSI_FLAGS) by combining bit-wise flags for different tests. A value of *Q* = 0 means that the estimate passed all metrics. Failures are identified by cumulatively increasing the value of *Q* for each quality assurance test that failed. *Q* is increased by 1 (figure-of-merit, FOM, failure), 2 (de-spike fraction failure), 4 (*ε* ratio failure), 8 (de-spike iteration failure), or 16 (variance resolution failure). The FOM provides a measure of how closely a spectrum agrees with the model spectrum over the range of wavenumbers that is used for the estimation of *ε* (equation 22 in Lueck *et al*.^2^), and a spectrum with FOM < 1.4 is recommended. In the quality control of our benchmark data, we used a more stringent condition of FOM < 1.15. Failures due to other user-defined criteria can be assigned values of 32, 64, 128 or 256. For example, a value of *Q* = 5( = 1 + 4) means that the dissipation estimate fails both the FOM and *ε* ratio tests. The final dissipation estimate (EPSI_FINAL) is the arithmetic average of the EPSI values that satisfy all quality-assurance criteria. If the dissipation estimates from a pair of probes disagree (*Q* = 4), the smaller rate is recommended as the final estimate, provided that it has an acceptable FOM. A description of the quality control metrics and their implementation is not within the scope of this paper and the reader can refer to the methods paper^2^ for details.

### Faroe bank channel – a deep overflow

**Fig. 2:**
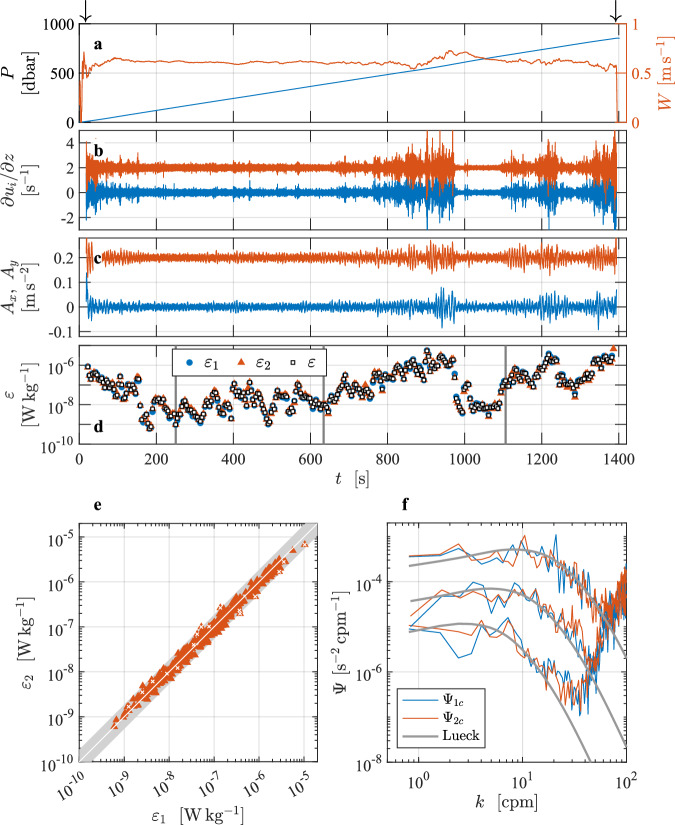
Overview of the vertical profile collected in the Faroe Bank Channel overflow^3^. Time series of (**a**) pressure, *P* (blue), and profiling speed, *W* (red). Time is in seconds since the start of the data file. (**b**) The cleaned (de-spiked) and high-pass filtered signals from shear probe 1 (blue) and 2 (red, offset by 2 s^−1^), and (**c**) accelerations *A*_*x*_ (blue), *A*_*y*_ (red, offset by 0.2 m s^−2^). Arrows in panel (**a**) mark the start and end of the selected section. (**d**) The rates of dissipation for the selected section for probe 1 (blue circles), probe 2 (red triangles), and the average of the estimates that passed quality assurance tests (black squares). (**e**) Scatter plot of *ε*_1_ and *ε*_2_ from the probe pair. Estimates failing the quality-assurance criteria are marked by white crosses. Gray band is the statistical uncertainty^2^ bounded by a factor of exp(2.77 × *σ*_ln*ε*_). (**f**) Clean wavenumber spectra from shear probe 1 (blue) and probe 2 (red) for three examples of quiescent, moderate and energetic dissipation estimates with 9.7 × 10^−10^ W kg^−1^, 1.1 × 10^−8^ W kg^−1^ and 1.6 × 10^−7^ W kg^−1^. The corresponding model spectra after Lueck^2^ are shown in gray. Times of the selected examples are marked by vertical gray lines in (**d**).

This dissipation profile^3^ is from the Faroe Bank Channel overflow, which is a bottom-attached, turbulent overflow plume of dense and cold water entering into the North Atlantic^8,9^ (Fig. 2). This profile was presented as a best practice example in Lueck *et al*.^2^, but not described in detail. The profile was collected using the tethered free-fall VMP2000 profiler (serial number 9, Rockland Scientific, Canada) on 10 June 2012 from the Research Vessel *Haakon Mosby*. The water depth is about 860 m. The dissipation rate was measured using two shear probes installed orthogonal to each other. Other sensors on the instrument were a fast-response FP07 thermistor, a Sea-Bird Electronics (SBE) microconductivity sensor, a 3-axis accelerometer, a magnetometer, and a pumped SBE conductivity-temperature package. The turbulence sensors were protected by a probe guard. The VMP sampled the signal plus signal derivative from the thermistor, microconductivity, and pressure transducer, and the derivative from the shear signals. The turbulence and acceleration channels were sampled at a rate of 512 s^−1^, while the other channels were sampled at 64 s^−1^. Data were transmitted to a shipboard data acquisition system. The instrument was deployed from the side of the vessel (drifting away from the profiler) using a hydraulic winch with a line-puller system, allowing it to fall freely at a nominal fall rate of about 0.6 m s^−1^.

When preparing the L2 time series, the shear probe and accelerometer time series are high-pass filtered using a cutoff frequency of 0.25 Hz, which corresponds to one-half of the inverse of the FFT length that is used for the analysis in L3. The shear data are cleaned using a first-order, low-pass, Butterworth filter with a cutoff frequency of 0.5 Hz and a threshold of 8 (the ratio of absolute shear to the smoothed absolute shear) using the method described in Lueck *et al*.^2^. The speed of profiling is estimated from the fall rate, *W*, which is calculated from the rate of change of pressure and smoothed with a low-pass filter with a cut-off frequency of 0.5 Hz that was applied both forwards and backwards. A section is extracted from the record when *W* was larger than 0.4 m s^−1^ (80% of estimated minimum fall rate) and when the depth exceeded 10 m to avoid ship effects. At the typical fall rate, the high-pass filtering applied in L2 suppresses the signals at vertical scales larger than the profiler length of about 2 m. Shear spectra are estimated using record lengths (diss_length_sec) of 8 s and FFT lengths (fft_length_sec) of 2 s that are cosine windowed and overlapped by 50%. Vibration-coherent noise is removed using the Goodman method^10^. The frequency spectra are converted to wavenumber spectra using the average fall rate for each spectrum. All dissipation estimates are smaller than 1 × 10^−5^ Wkg^−1^ and, therefore, they were obtained using the spectral integration method. Successive dissipation estimates are overlapped by 50%, i.e., overlap_sec = 4 s. The fraction of the shear variance not resolved within the integration limits is corrected by using the Lueck model spectrum. The quality assurance is done conforming with the ATOMIX recommendations^2^.

### Haro strait – a tidal channel

**Fig. 3:**
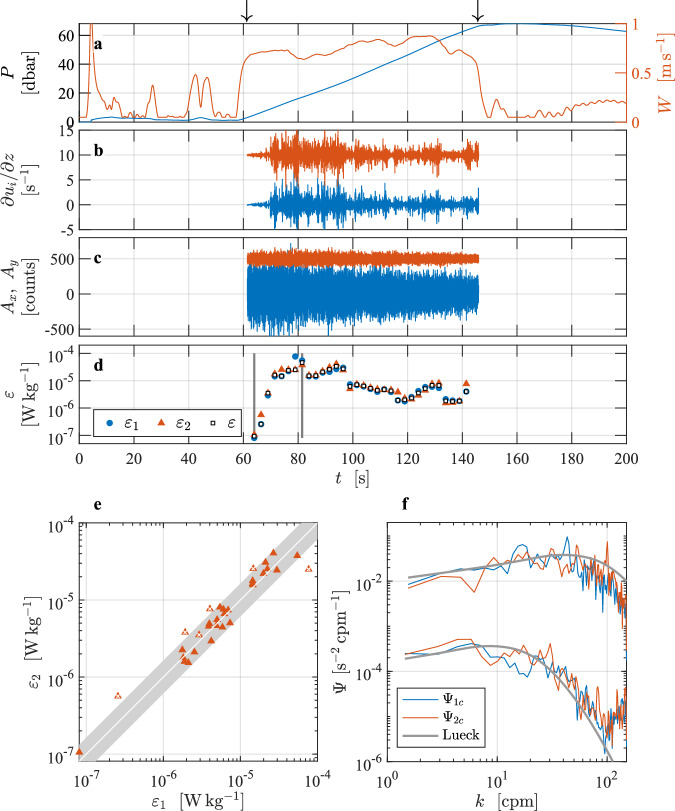
Overview of the vertical profile collected in a tidal channel in Haro Strait^4^ with a VMP-250 turbulence profiler. Panels are similar to Fig. 2. Records are offset by 10 for shear (**b**) and by 500 units for acceleration (**c**). The reference spectra shown in (**f**) are for *ε* = 4.6 × 10^−5^ W kg^−1^ (upper curve) and 9.3 × 10^−8^ W kg^−1^ (lower curve).

This turbulence profile^4^ was taken in a side channel adjacent to Haro Strait on the east coast of Vancouver Island, British Columbia, Canada, on 19 October 2016 (Fig. 3). The instrument was a VMP-250-IR (internally recording and serial number 215) vertical profiler from Rockland Scientific, Canada. It was configured to collect data while descending, with two shear probes oriented to measure two orthogonal components of the vertical shear of horizontal current, one FP07 thermistor, and two vibration sensors, all of which were anti-alias low-pass filtered at 98 Hz and sampled at a rate of 512 s^−1^. Additional sensors include conductivity and temperature sensors (JFE Advantech, JAC), a two-axis inclinometer, and a pressure transducer, which were all sampled at a rate of 64 s^−1^. There was a guard that protected the sensor array on the leading edge of the vertical profiler. The speed of profiling was deduced from the rate of change of pressure which was smoothed using a first-order low-pass filter with a cutoff frequency of 0.45 Hz, that was applied forwards and backwards. All speeds smaller than 0.05 m s^−1^ were set to this value to avoid infinity values in the conversion to physical units. This does not affect the data in the analyzed section, because it uses only speeds faster than 0.5 m s^−1^.

The water depth was approximately 80 m and the water column was quite turbulent below 10 m due to a tidal current exceeding 1 m s^−1^. The typical speed of profiling was 0.7 m s^−1^ (Fig. 3a, red). The profiler was tethered with a nearly neutrally buoyant line and deployed off the stern of a small fishing vessel that was converted for scientific research (the Research Vessel *Strickland*). The profiler lingers just below the surface for 90 s, descends to about 65 m, and is then pulled back to the surface by its tether line (Fig. 3a). During the descent, the line was paid out fast enough to maintain a few coils of slack near the surface. This allowed the instrument to move with minimal disturbance by the slight vertical motion of the deployment vessel. A section (or profile) was extracted based on a minimum pressure of 3 dbar and a minimum descent speed of 0.5 m s^−1^, when both lasted for at least 20 s (identified by arrows in Fig. 3a).

The shear-probe and vibration signals in the extracted section were high-pass filtered at 0.4 Hz. The shear data were de-spiked using a threshold of 8 (the absolute to the smoothed absolute shear ratio), a smoothing low-pass filter of 0.5 Hz, and a duration removal of 0.04 s, as described in Lueck *et al*.^2^. If the initial estimate of the rate of dissipation, based on a spectral integration to 10 cpm (cycles per meter) was smaller than 1.5 × 10^−5^ W kg^−1^, the dissipation estimate was made using the method of spectral integration. For larger initial rates, the rate of dissipation was estimated by a fitting of the spectrum in the inertial subrange.

**Fig. 4:**
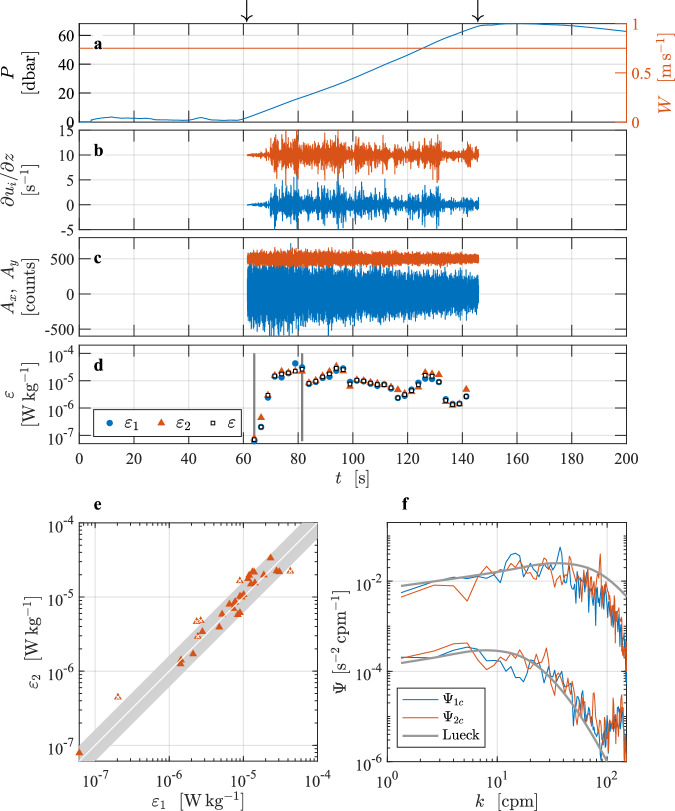
Similar to Fig. 3 but using a constant speed of profiling of *W* = 0.75 m s^−1^. This changes the magnitude of the shear signals and makes the reference spectra (in panel f) for the largest and smallest rates equal to 2.6 × 10^−5^ and 7.0 × 10^−8^ W kg^−1^, respectively.

There is a second file that was derived from the same raw data file but was converted into physical units using a constant speed of profiling equal to *W* = 0.75 m s^−1^ (Fig. 4). The reason for converting the data into physical units using a constant speed is that there are very strong up- and down-drafts in the water column. In the presence of strong vertical motions, the rate of change of pressure does not give a good measure of the speed of streaming over the shear probes. For example, if there is an up-draft equal to the nominal descent rate of the profiler, the rate of change of pressure would be zero even though there is flow past the sensor equal to its nominal rate of descent. Using a constant speed changes the conversion of shear into physical units and also the estimated rate of dissipation because these scale as *W*^2^ and *W*^4^ (Lueck *et al*.^2^). A constant speed, equal to the typical or average speed of an instrument, may provide a better estimate of the rate of streaming past the shear probes than the rate of change of pressure because this profiler (and probably many others) adjusts its speed relative to its surrounding fluid in a distance shorter than its length. This is inferred from its initial acceleration after its tether is released.

### Rockall trough – a deep bottom-boundary layer

**Fig. 5:**
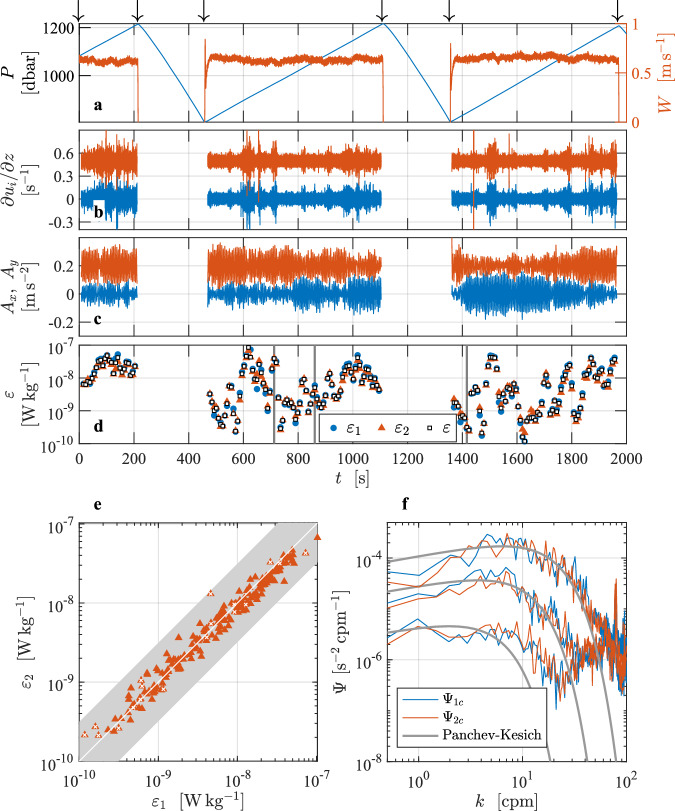
Overview of the Rockall Trough data^5^ collected with the epsilometer. Panels are similar to Fig. 2. Three sections (between arrows) are defined as the portions of the record with a steady fall rate exceeding 0.2 m s^−1^. Records are offset by 0.5 s^−1^ for shear (**b**) and by 0.2 m s^−2^ for accelerations (**c**). The reference spectra shown in (**f**) are for *ε* = 3.9 × 10^−8^ W kg^−1^ (upper curve), *ε* = 5.1 × 10^−9^ W kg^−1^ (middle curve), and 3.1 × 10^−10^ W kg^−1^ (lower curve). The reference spectrum used in this data set is from Panchev-Kesich^2^.

These microstructure profiles^5^ were collected along the slope of a canyon south-west of Ireland, inside the Rockall Trough, using the *epsilometer*–a microstructure turbulence sensor built by the Multiscale Ocean Dynamics group at the Scripps Institution of Oceanography^11^. The data were collected in July 2021 as a part of the “Boundary layer turbulence experiment project”. The dissipation rates *ε* were measured using two airfoil shear probes with their sensitive direction aligned with each other. In addition to the shear data, the analog electronics of the epsilometer sample at 320 s^−1^ measurements from two FP07-thermistors and a three-axis piezo-accelerometer. The epsilometer-controller collects data from a Sea-Bird Scientific SBE49 CTD (16 s^−1^), an inertial measurement unit (Vector Nav, 40 s^−1^) and an altimeter (1 s^−1^). The epsilometer was mounted on its custom-designed vehicle: the epsifish. The epsifish was operated from a custom fast CTD winch from a moving research vessel. The fast CTD winch can power the epsifish and receive the data back while unspooling with just enough slack to ensure a free-fall downcast. With an altimeter mounted on the epsifish, this winch repeats highly detailed profiles within a few meters of the seafloor (or a maximum of 2200 m depth). The epsilometer fall rate is about 0.6 m s^−1^ (Fig. 5a). The pressure data *P* of the SBE49 are too noisy for a direct estimate of the time derivative of the pressure. In practice, we apply a low-pass filter with a 1 Hz cut-off frequency to reduce the pressure noise. The Rockall Trough benchmark record consists of 3 successive sections of steady fall rate faster than 0.2 m s^−1^. The epsilometer records successive one-hour-long files. Consequently, the raw files are not organized per profiles but as time series. Hence, a file and may start in the middle of a profile such as the case in the first section of this dataset. The epsilometer does not have a *differentiator* circuitry, and thus, the shear probes provide time series of micro-scale ocean velocity. The shear time series are obtained by applying a first-order differentiation in time during post-processing. These time series are high-pass filtered at 0.1 Hz, and a de-spiking procedure is done within a 500 ms window using the Matlab function “filloutliers”. Shear spectra are estimated using record lengths of 5120 samples (diss_length_sec = 10.4 s), which are partitioned into FFT segments of 1024 samples (fft_length_sec = 2.08 s) that are Hanning(cosine)-windowed and overlapped by 50%. Vibration-coherent noise is removed using the Goodman method^10^ using only the third axis of the accelerometer, which is parallel to the sensitive axis of the shear probes. The frequency spectra are converted to wavenumber spectra using the average fall rate for each spectrum. All but two dissipation estimates are smaller than 1 × 10^−5^ W kg^−1^, and therefore, they are obtained using the spectral integration method. The two exceptions are obtained using a spectral fit in the inertial subrange but they are discarded because they fail to pass the quality-assurance metrics. Successive dissipation estimates are overlapped by 50%, i.e., overlap_sec = 5.2 s. The differentiation to obtain shear mentioned above acts as a filter, and its response must be corrected in the wavenumber spectra of shear and related sensors before estimating *ε*. Similarly, the transfer function of a charge amplifier integrated into the epsilometer’s analog electronics impacts the shape of the shear spectra. This transfer function is provided in the netCDF file (variable CA_TF). These corrections–the differentiation and the charge amplifier transfer function–are applied to the vertical wavenumber spectra in L3. The user must divide the raw shear spectrum by the corresponding transfer function to obtain the spectra published in the dataset in L3. Estimates of *ε* are obtained using the iterative method^2^ using the Panchev-Kesich model spectrum.

### Baltic sea – a quiescent profile

**Fig. 6:**
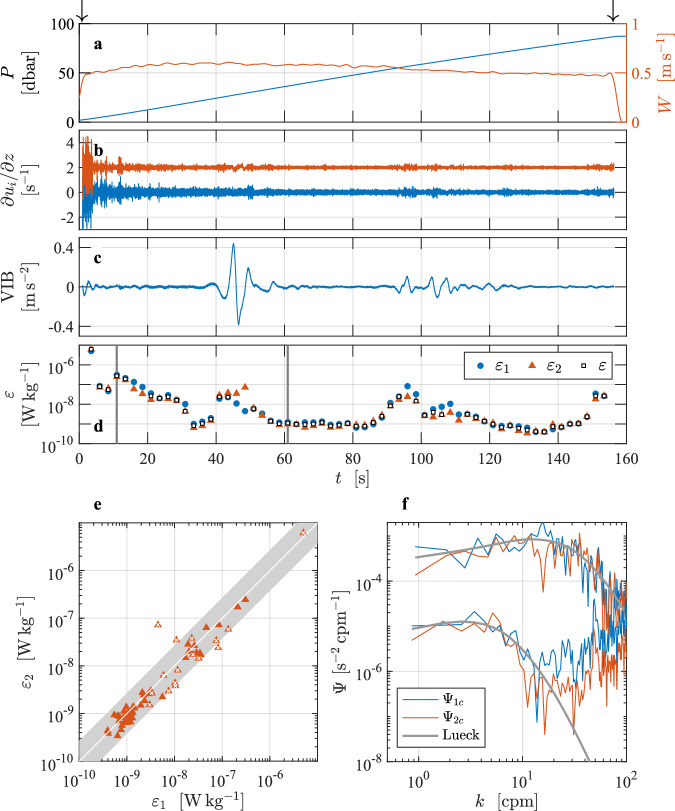
Overview of the Baltic Sea, Bornholm Basin data^6^ sampled with a MSS90-L. Panels are similar to Fig. 2, except for the vibration data VIB instead of acceleration in (**c**). The reference spectra shown in (**f**) are for *ε* = 2.8 × 10^−7^ W kg^−1^ (upper curve) and 1.1 × 10^−9^ W kg^−1^ (lower curve).

The Baltic Sea profile^6^ was taken in September 2008 in the Bornholm Basin^12^ from the Research Vessel *Poseidon*. The oceanographic context and a detailed analysis of the data collected during this cruise are given in van der Lee and Umlauf^12^. The total depth was about 85 m. The instrument used was a MSS-Microstructure profiler with the serial number 38, produced by Sea & Sun Technology, Germany (Fig. 6). It was equipped with two randomly oriented shear probes, one FP07 thermistor, and one PT100 temperature sensor and a conductivity cell for precision temperature and salinity measurements. The probe was also fitted with a vibration sensor, which is similar in design to a shear probe sensor, except that the airfoil is replaced by a small mass and is mounted inside the probe. The probe was connected with a cable to a ship-board data acquisition system and was operated with an electrical winch from the stern of the ship. During the profiling, the ship slowly moved along a transect with a speed of approximately 0.5 m s^−1^. The transect was oriented such that wind and surface currents came from the bow, such that the loosely tethered cable was always at a safe distance to the ship’s propeller. Using a sensor protection cage, it was possible to obtain nearly full-depth vertical profiles to within 0.1 m above the seafloor, with a descent speed of approximately 0.5 m s^−1^.

All sensors were sampled at a rate of 1024 s^−1^ with 16 bit resolution. Similar to the epsilometer, the MSS does not measure the time-derivative of the shear-probe signal. To obtain the velocity shear, the shear probe data is differentiated in time by applying a first-difference filter during post-processing. The shear probe and vibration time series are high-pass filtered with a cutoff frequency of 0.15 Hz and de-spiked according to Lueck *et al*.^2^ using a threshold of 8, a smoothing low-pass filter of 0.05 Hz, and a duration removal of 0.04 s. The dataset consists of one section, which was chosen manually by selecting the data during which the probe descended with *W* greater than 0.4 m s^−1^. Shear spectra were estimated using record lengths of 5 s and FFT lengths of 2 s. The FFT segments are cosine windowed and overlapped by 50%. Vibration-coherent noise is removed using the Goodman method^10^. Both sensors measure an arbitrary horizontal velocity component that is used to derive the vertical shear. The individual dissipation rates of both sensors agree well, which is expected due to the isotropic nature of the shear velocity microstructure^2^. All dissipation estimates are smaller than 1 × 10^−5^ W kg^−1^ and, therefore, they were obtained using the spectral integration method. Successive dissipation estimates are overlapped by 50%. The dissipation rates of the profile are frequently at the noise level of the instrument. Elevated *ε* occurs in the well-mixed upper water column, within the halocline in 50 m depth and near the seafloor.

### Minas passage – a moored turbulence profile

**Fig. 7:**
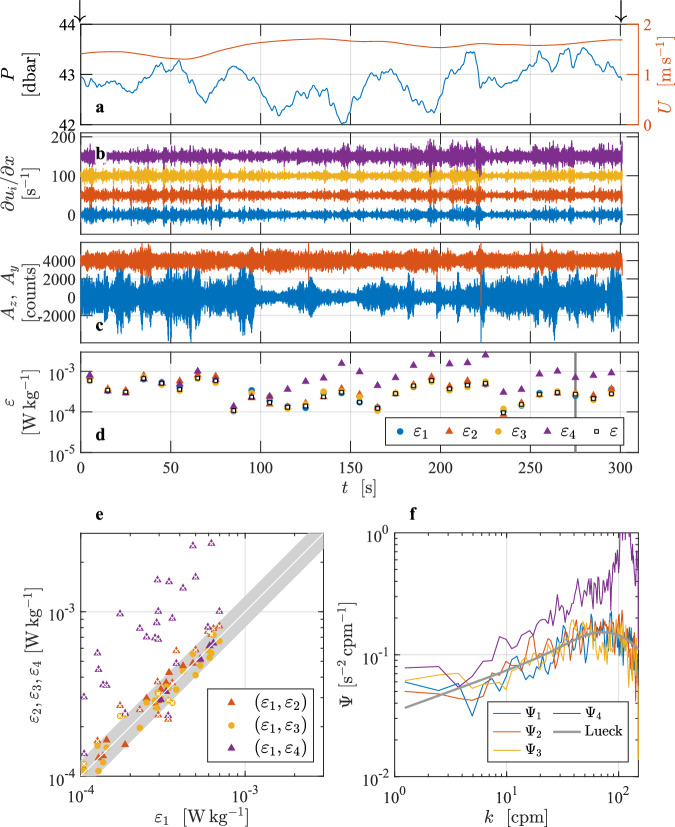
Overview of data collected from a mooring that is 15 m above the bottom in a swift tidal channel in Minas Passage^7^. Panels are similar to Fig. 2. This is a horizontal profile in which the streaming is provided by the tidal current (**a**, red). The instrument carries four shear probes, two of which measure the horizontal gradient of vertical velocity (**b**, blue and yellow), while the other pair measure the horizontal gradient of lateral velocity (**b**, red and magenta). Shear probe records are offset by 50. Vibrations are measured in the vertical (**c**, blue) and the lateral (**c**, red, offset by 4000 units) directions. The reference spectrum shown in (**f**) is for *ε* = 2.8 × 10^−4^ W kg^−1^.

An example of a horizontal profile is provided^7^ using data collected in Minas Passage from a bottom-anchored Nemo mooring at an elevation of 15 m above the bottom (Fig. 7). This record was collected with a modified MicroRider-1000 (Rockland Scientific, Canada) that was mounted into the leading edge of a streamlined float^13^ on 10 September 2016. The water depth at low tide is approximately 60 m. The Nemo float uses a bridle, a swivel, and tail fins to orient itself horizontally and to point into the oncoming flow. The MicroRider carried four shear probes. Two measured ∂*w*/∂*x* while the other pair measured ∂*v*/∂*x*, where *x* is directed into the streaming, and *w* and *v* are nominally directed vertically and to port (laterally), respectively. The instrument also carried two piezo vibration sensors and one FP07 thermistor. The foregoing were all sampled at a rate of 2048 s^−1^ and were anti-alias low-pass filtered at 392 Hz. Other sensors include a three-axis accelerometer, a three-axis rotation sensor, a three-axis magnetometer, and a pressure transducer. These were sampled at a rate of 256 s^−1^. The speed of profiling was measured by a three-axis acoustic current meter that had its sampling volume about 1 m aft and 0.6 m above the shear probes. It was sampled at a rate of 4 s^−1^. The shear probes are separated from each other by 0.044 m and 0.025 m in the lateral and vertical directions, respectively.

The L1 data is an arbitrary 300 s record from the deployment, and the entire record satisfies the criteria of selecting a section. When preparing the section in L2, the shear-probe and vibration data were high-pass filtered at 1 Hz. The shear data were de-spiked using a threshold of 8, a smoothing low-pass filter of 1 Hz, and a duration removal of 0.04 s, as described in Lueck *et al*.^2^. During this 300 s sample, the currents and hence, the speed of profiling, ranged from 1.3 to 1.7 m s^−1^ (Fig. 7a, red). The fluctuations of the magnitudes of the signal reported by the two probes that measure ∂*w*/∂*x* and one of the two probes that measure ∂*v*/∂*x* agree with each other (Fig. 7b, lower three traces). However, the signal from the fourth probe ((Fig. 7b, upper trace) is clearly stronger in the second half of this section. It is highly likely that the fourth probe snagged a piece of seaweed that caused it to produce erroneous signals. We cannot be certain that this is the case because the anomalously strong signal ceased about an hour later and the instrument came to the surface clean after its two-week deployment. However, on other recoveries there were seaweeds wrapped around the base of the shear-probes. Vibrations are stronger in the vertical than in the lateral directions (Fig. 7c). All dissipation estimates were made by fitting the shear spectra in the inertial subrange because all estimates are much larger than 1 × 10^−5^ W kg^−1^ (Fig. 7d).

## Data Records

The datasets are available at the British Oceanographic Data Centre of the National Oceanography Centre. The direct links to the datasets are provided in Table 1. The data records are stored in Network Common Data Form (netCDF) version 4 files in the ATOMIX format (Tables 3–6). Each file includes four hierarchical groups, corresponding to the four processing levels. The group names are L1_converted, L2_cleaned, L3_spectra and L4_dissipation. All data files contain the required metadata (Table 7) and optional metadata (Table 8) as global attributes using Climate and Forecast (CF 1.6), Attribute Convention for Data Discovery (ACDD 1.3) and ATOMIX 1.0 conventions. All data files contain the required and highly recommended variables, as well as the optional variables when available, of the four processing levels listed in Tables 3–6. The stored variables are provided with associated attributes standard_name, units and long_name. An additional comment attribute is provided for some variables when clarification is needed.

An overview of the data collection sites is given in Fig. 1. The datasets are chosen to span a wide range of depth, flow dynamics and sampling strategies using different platforms and instruments. The data files from the Faroe Bank Channel^3^ (10.5285/05f21d1d-bf9c-5549-e063-6c86abc0b846), Haro Strait^4^ (10.5285/0ec16a65-abdf-2822-e063-6c86abc06533) and Minas Passage^7^ (10.5285/0ec17274-7a64-2b28-e063-6c86abc0ee02) were recorded with Rockland Scientific instruments and additionally provide the instrument setup file in the metadata. The dataset from the Baltic Sea^6^ was measured by a Sea & Sun Technology instrument and is provided at 10.5285/0e35f96f-57e3-540b-e063-6c86abc06660. The dataset from the Rockall Trough^5^ was measured by an Epsilometer developed at Scripps Institution of Oceanography and is provided at 10.5285/0ebffc86-ed32-5dde-e063-6c86abc08b3a.

## Technical Validation

Dissipation rate measurements in the Faroe Bank Channel^3^ section cover four orders of magnitude from 10^−9^ W kg^−1^ to 10^−5^ W kg^−1^ (Fig. 2d), all exceeding the lowest-detection level of about 10^−10^ W kg^−1^ (the so-called the noise level) for *ε* from this instrument. Dissipation rate estimates from both probes agree to within their expected statistical uncertainty^2^ of exp(2.77 × *σ*_ln*ε*_) (Fig. 2e). The value of *σ*_ln*ε*_, the variable name EPSI_STD in the data file, is the expected standard deviation of the logarithm of the dissipation estimate. For pairs of dissipation estimates, *σ*_ln*ε*_ spans 0.14 to 0.33, resulting in the uncertainty factor of 1.5 to 2.5 (on the figure, the gray band is shown for the mean value of *σ*_ln*ε*_ = 0.23 over all estimates, resulting in a factor of 1.86). Wavenumber spectra from three examples of quiescent, moderate and energetic dissipation estimates with 9.7 × 10^−10^ W kg^−1^; 1.1 × 10^−8^ W kg^−1^; 1.6 × 10^−7^ W kg^−1^ from both probes agree with each other and follow the form of the Lueck spectrum. We have 342 dissipation estimates per probe. Most estimates are of high quality and pass the quality-assurance tests (*Q* = 0). When the estimates from both probes are accepted, the final dissipation estimate is the average over both probes. When the estimate from one of the probes fails the tests, the final estimate comes from the other probe. In total, 9 estimates from each probe failed the tests. We applied a stringent threshold of FOM = 1.15. Of those, 7 estimates from probe 1 and 5 from probe 2 failed the FOM test (*Q* = 1), and 2 from probe 1 and 4 from probe 2 failed the dissipation ratio test (*Q* = 4). Only two estimates from both probes simultaneously failed the test (all because they exceeded the FOM criterion), resulting in 340 EPSI_FINAL estimates. For these depth (time) ranges when both probes fail the quality-assurance tests (near 100 and 600 dbar), no value is reported.

In the Haro Strait tidal channel dataset^4^, 15 of the 64 estimates were made by way of fitting in the inertial subrange. The minimum and maximum rates of dissipation (EPSI_FINAL) are 9.3 × 10^−8^ and 4.6 × 10^−5^ W kg^−1^, respectively (Fig. 3d, their spectra are shown in Fig. 3f). Six of the estimates fail the quality assurance tests – five for a dissipation ratio failure and one because its figure of merit exceeded 1.15. The fall-rate variations that are based on the rate of change of pressure, (Fig. 3a, red) are unusually large and this is undoubtedly due to large up- and down-drafts in the channel due to the vigorous turbulence generated by bottom friction. An alternative processing using a constant speed of profiling could be considered as exemplified in Fig. 4.

Of the 181 estimates of *ε* per shear probes collected by the epsilometer in the Rockall Trough data set^5^, a total of 20 samples failed the FOM≤1.15 criteria and, consequently, are flagged with *Q* = 1. For both shear probes, a couple of *ε* estimates (*ε* index = 115, 116) are flagged with *Q* = 4 and *Q* = 5 for probe 1 and probe 2, respectively. Similarly to the other datasets, *Q* = 4 indicates these *ε* failed the dissipation ratio test and *Q* = 5 (i.e., *Q* = 4 + 1) indicates that these *ε* failed both the dissipation ratio test and the FOM≤1.15 criteria. This is due to the presence of a spike in the shear time series of probe 2 soon after 1400 s (Fig. 5). The de-spiking routine could not correct for that spike. All but two of the 64 dissipation rate estimates were smaller than 1 × 10^−5^ Wkg^−1^, and their estimation was made using spectral integration. The two exceptions were estimated using the inertial subrange fitting method and were identified in the L4 group with a METHOD value of 1.

All 61 dissipation rate estimates of the Baltic Sea dataset^6^ were below 10^−5^ W kg^−1^ and were, thus, made by spectral integration. The profile is representative of the low energy environment of the deep basins of the Baltic Sea, with a majority of the dissipation rates at the instrument’s noise level of 1 × 10^−9^ W kg^−1^. The relatively large noise level, in comparison to other instruments, is a consequence of the light mass of the profiler and the robust protection cage that allows the probe to touch the seafloor without damage to the sensors. Eight out of 61 estimates fail the quality assurance tests for both probes. A failure was either by exceeding the FOM limit of 1.15 (*Q* = 1, which occurred 4 and 5 times for shear probe 1 and 2, respectively), by failing the dissipation ratio test (*Q* = 4, which occurred 7 and 2 times for probe 1 and 2, respectively), or both (*Q* = 5, which occurred 2 and 4 times for probe 1 and 2, respectively). During parts of the section, substantial vibrations degenerate the quality of the shear probe signal and the number of quality assurance failures increases.

In the turbulent waters of Minas Passage^7^, all dissipation rate estimates were obtained from a fit of the shear spectra in the inertial subrange. The rate of dissipation estimates from the four shear probes agree for the first 100 s of this profile (Fig. 7d), but the rate reported by probe 4 that measures ∂*v*/∂*x* exceeds the others by as much as a factor of 10 during the remainder of this profile (Fig. 7d, magenta triangles). More than two-thirds of the dissipation estimates from probe 4 fail a quality-assurance metric (Fig. 7e, white crosses in the magenta triangles), which is not surprising given that a piece of seaweed probably wrapped itself around this probe. Failures are due to a large ratio of the estimated rate of dissipation of this probe relative to the others, and due to a large FOM because of the anomalous shape of the spectra from this probe (Fig. 7f, magenta line). Spectra from the third last dissipation estimates (marked with a gray bar in Fig. 7d) agree for probes 1 through 3 and these spectra also agree with the model spectrum (gray line). However, the spectrum from the fourth probe is higher than the spectra from the other probes by more than a factor of 2 (Fig. 7f, magenta) and it has a shape that differs noticeably from the model spectrum. Vibrations are stronger in the vertical than in the lateral directions (Fig. 7c). This is quite common. The level of vibration depends on the mechanical stiffness of the components in an instrument. The electronics are usually mounted on a frame internal to a pressure case, and the aspect ratio of the dimensions of the frame often makes them stiffer in one direction compared to the orthogonal one.

## Usage Notes

The benchmark datasets (Table 1) will serve as a valuable resource for users to evaluate their routines and allow for platform-independent analysis of shear probe data once the L1 data are provided. The user can benefit from the benchmarks, which provide practical examples of the recommended data format and the metadata. The provided code^14^ that is used to produce the figures in this paper may serve as an example of how to load and handle different levels of data. Users can then analyze data from their desired level, such as starting with L1, selecting sections of cleaned time series from L2, or using corrected shear spectra from L3. The homogeneous data format allows easy and transferable workflow from one dataset to the other, streamlining reprocessing and inter-comparison of data. We strongly recommend the practitioners submit and archive their shear probe data in the ATOMIX format described in this paper.

The user can test their routines for dissipation estimates from shear probes against benchmark datasets before analyzing and publishing their science and data. If the user does not have data, the L1 data provided in the benchmarks offer an opportunity to develop and test routines. We would strongly encourage that a statement is included in their study that their methods conform to the ATOMIX best practices and successfully reproduce the benchmark estimates, offering confidence in their estimates for the peer-reviewers and the readers. While established practitioners could still produce quality data without implementing the ATOMIX guidelines, independent tests of the benchmarks with their routines and providing feedback would strengthen and further improve the development of best practices for the ocean mixing community. With the availability of benchmark datasets in a uniform format, we expect the adoption of the ATOMIX recommendations on an increasingly larger scale and a standardized format for archiving.

The consequences and implications of various choices of data processing parameters can be tested. For instance, the user could test the influence of poor profiling speed estimates in the energetic tidal channel. Would it be more appropriate to convert the shear-probe data into physical units using a constant speed of profiling? The average speed of profiling in the selected section is 0.75 m s^−1^. Loading the data from L2 (using the cleaned shear data from the section), but imposing a constant profiling speed, the user can recalculate the spectra and obtain dissipation estimates. In the tidal channel dataset^4^, we also supply a second file named VMP250_TidalChannel_024_cs (Fig. 4), in which we used a constant profiling speed of 0.75 m s^−1^. All other processing parameters remained unchanged from the processing applied to the file that uses the rate of change of pressure to determine the fall rate of the profiler. Using a constant speed of profiling reduced the lowest rate of dissipation slightly to 7 × 10^−8^ W kg^−1^. However, a constant speed reduced the largest rate by almost a factor of two to 2.6 × 10^−5^ W kg^−1^ (Fig. 4f). The quality assurance failures remained the same.

The spectra provided in the benchmark L3 records make the application of alternative dissipation estimate methods accessible. The user can experiment with the sensitivity of dissipation rate estimates to (i) cleaned versus measured shear spectra, (ii) alternative model spectra, (iii) different choices of wavenumber range for analysis, and (iv) spectral fitting versus integration methods. A demonstration of this can also be found in Lueck *et al*.^2^ where the Faroe Bank benchmark data^3^ in L3 were used to obtain alternative estimates by fitting the spectra in the inertial subrange.

## Data Availability

Matlab computer software used to read the data and produce the figures from the netCDF files, together with a Python script to check the required content of an ATOMIX netCDF file is available from the ATOMIX Shear Probes GitHub repository (https://github.com/SCOR-ATOMIX/ShearProbes_BenchmarkDescriptor). The present paper is based on version 1.0, available from Fer *et al*.^14^, at 10.5281/zenodo.10610150.
